# Association of p53 with Neurodegeneration in Parkinson's Disease

**DOI:** 10.1155/2022/6600944

**Published:** 2022-05-11

**Authors:** Qiang Luo, Wei Sun, Yi-Fan Wang, Ji Li, Da-Wei Li

**Affiliations:** ^1^Department of Personnel, Affiliated Hospital of Beihua University, Jilin 132011, China; ^2^Department of Neurology, Shenzhen Sammi Medical Center, Shenzhen 518000, China; ^3^Department of Neurology, Affiliated Hospital of Beihua University, Jilin 130021, China

## Abstract

p53 is a vital transcriptional protein implicated in regulating diverse cellular processes, including cell cycle arrest, DNA repair, mitochondrial metabolism, redox homeostasis, autophagy, senescence, and apoptosis. Recent studies have revealed that p53 levels and activity are substantially increased in affected neurons in cellular and animal models of Parkinson's disease (PD) as well as in the brains of PD patients. p53 activation in response to neurodegenerative stress is closely associated with the degeneration of dopaminergic neurons accompanied by mitochondrial dysfunction, reactive oxygen species (ROS) production, abnormal protein aggregation, and impairment of autophagy, and these pathogenic events have been implicated in the pathogenesis of PD. Pathogenic p53 integrates diverse cellular stresses and activate these downstream events to induce the degeneration of dopaminergic neurons; thus, it plays a crucial role in the pathogenesis of PD and appears to be a potential target for the treatment of the disease. We reviewed the current knowledge concerning p53-dependent neurodegeneration to better understand the underlying mechanisms and provide possible strategies for PD treatment by targeting p53.

## 1. Introduction 

Parkinson's disease (PD) is a common neurodegenerative disorder caused by the selective and progressive loss of dopaminergic neurons in the substantia nigra (SN) of the midbrain and depletion of dopamine neurotransmitter in the striatum [1]. The etiology responsible for the progressive degeneration of dopaminergic neurons remains unclear. However, multiple pathogenic events, including mitochondrial dysfunction, oxidative stress, abnormal protein aggregation, and impairment of mitophagy, have been documented to be mechanistically linked to the pathogenesis of PD [2–5]. p53 is known to be an essential apoptotic inducer and becomes activated in response to diverse cellular stresses. Pathogenic p53 integrates the cellular stresses to trigger the death of different cell types, including dopaminergic neurons [6, 7]. Studies in cellular models of PD have demonstrated that p53 levels and activity are substantially increased, and these changes are closely associated with dopaminergic neuron death in neurodegenerative conditions. The high levels of p53 were also observed in the brains of PD patients as well as PD animal models, supporting the link between p53 activation and the degeneration of dopaminergic neurons in PD [8]. The activation of p53 induces neurodegeneration through diverse cell death pathways, including mitochondrial dysfunction, mitochondrial Ca^2+^ overloading, reactive oxygen species (ROS) production, abnormal protein aggregation, and impairment of mitophagy [9, 10]. p53 brings together diverse pathogenic signals to initiate downstream pathogenic events and consequent neurodegeneration; thus, it plays a central role in the pathogenesis of PD and provides a potential target for therapeutic intervention of the disease. This article reviewed the involvement of apoptotic mediator p53 in pathogenic events associated with the loss of dopaminergic neurons and the underlying mechanisms responsible for p53-mediated neurodegeneration in PD.

## 2. Property of p53

p53 is a transcriptional protein encoded by the TP53 gene. It was initially described as a tumor suppressor. However, later studies revealed that p53 is a multifunctional protein involved in regulating numerous cellular processes by activating diverse downstream signal cascades [11–15]. Structurally, p53 contains five highly conserved domains: a central DNA-binding domain, an N-terminal transactivation domain, a proline-rich region, a tetramerization domain, and a C-terminal basic domain, which are associated with the transcriptional or posttranscriptional regulating function of p53 [16]. The DNA-binding domain is a primary functional domain of p53 that recognizes and binds to specific DNA sequences in target genes, triggering the transcription of sets of genes with diverse biological functions [17–19]. The C-terminal basic domain stabilizes the formation of p53-DNA complexes by inducing the conformational changes in the core DNA-binding domain. It is also a specific site for p53 posttranslational modifications including phosphorylation, acetylation, ubiquitination, methylation, SUMOylation, and neddylation, and these structural changes are closely associated with p53 stability and functional activity [20–25]. The N-terminal domain and the proline-rich region are correlated with p53 transcriptional activation, while the oligomerization domain contributes to the stability of p53-DNA complexes, thereby promoting p53 transcriptional function. Normally, p53 is an unstable protein that is continuously degraded by proteasomes. The murine double minute-2 (Mdm2) protein is known to be a major negative regulator that targets p53 for proteasomal degradation and inhibits its subcellular translocation by ubiquitinating p53 [26]. Phosphorylation of p53 at Thr377 and Ser378 decreases its acetylation and activity and facilitates its ubiquitination and degradation, while Ser15, Thr18, or Ser20 phosphorylation increases its ability to counteract ubiquitin-mediated protein degradation, promotes C-terminal acetylation and nuclear transport, and enhances its DNA binding and transcriptional activity [27–30]. Phosphorylation at Ser46 of cytoplasmic p53 activates its conformational change and mitochondrial translocation [31, 32]. p53 is a response gene that regulates the transactivation of many target genes involved in diverse biological processes. p53 activation-associated degeneration of dopaminergic neurons has been reported to be closely associated with the development of PD [10, 33].

## 3. p53 with Mitochondria

Mitochondria are multifunctional subcellular organelles that are essential for numerous cellular functions, including generation of cellular energy, intracellular Ca^2+^ homeostasis, ROS production, and activation of intrinsic cell death pathways [34–36]. Mitochondrial dysfunction has been implicated in a series of diverse diseases including PD and has been reported to be a central event in PD pathogenesis [37]. Activation of p53-mediated mitochondrial apoptotic changes and the subsequent cell death of dopaminergic neurons have been underlined in neurodegeneration [38]. Experimental and clinical studies have demonstrated that the levels and activity of p53 are highly increased in PD cellular and animal models as well as in the brains of PD patients, and these changes are closely associated with the dysfunction of mitochondria and the cell death of dopaminergic neurons [8]. p53 activation has a profound influence on mitochondrial integrity and function through transcription-dependent mechanisms and transcription-independent actions.

### 3.1. p53 and Mitochondrial ROS Production

**Figure 1:**
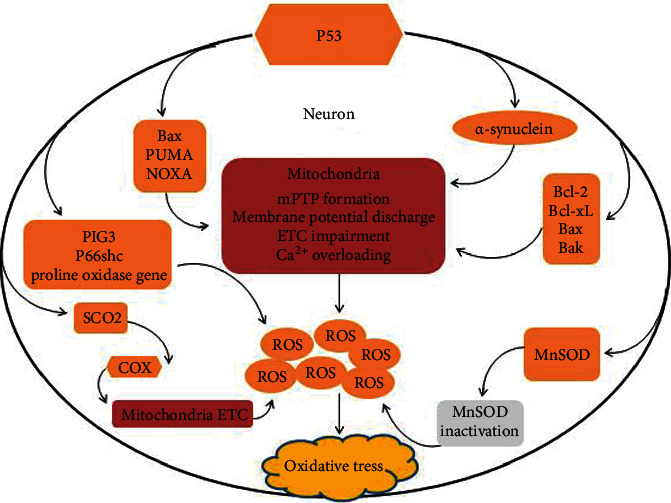
p53 activation in ROS production under irreversible stress conditions. p53 regulates cellular redox homeostasis through transcriptional action and modified expression of pro-oxidant and antioxidant proteins. p53 activation induces the expression of many proapoptotic proteins, such as Bax, PUMA, and NOXA, and facilitates Bax/Bak-mediated permeabilization of the outer mitochondrial membrane leading to the discharge of the membrane potential and ETC impairment followed by excessive ROS production. p53 also activates pro-oxidative genes including PIG3, proline oxidase, and p66shc to induce elevated levels of oxidative stress. The pro-oxidative activities of p53 also include the activation of expression of SCO2 gene, which is essential for regulating COX complex, the major site of oxygen utilization in the eukaryotic cells. The accumulation of cytosolic p53 protects *α*-synuclein from ubiquitin-mediated degradation inducing ROS generation and oxidative stress. *α*-Synuclein, in respone to cellular stress specifically targets mitochondria causing their profound alterations, including collapse of transmembrane potential, impairment of respiratory chain complexes, disturbance of mitochondrial Ca^2+^ homeostasis, and subsequent excessive ROS production. Accumulation of p53 in the mitochondrial matrix also contributes to oxidative damage to target cells by binding and inactivating the antioxidant MnSOD.

Oxidative stress is a pathogenic condition resulting from an imbalance between ROS production and cellular enzymatic and nonenzymatic antioxidative defenses. Oxidative damage to dopaminergic neurons has been considered as an essential pathogenic factor in the development of PD [39]. This is supported by the findings that the brain tissues of PD patients express high levels of oxidative products, including lipid peroxidation product 4-hydroxyl-2-nonenal (HNE), carbonyl modifications of soluble proteins, and DNA and RNA oxidation products 8-hydroxy-deoxyguanosine and 8-hydroxyguanosine [40–43]. Oxidative damage of dopaminergic neurons has also been observed in PD animal and cellular models, supporting the correlation of oxidative stress with the degeneration of dopaminergic neurons in PD [44–46]. Mitochondria are a primary intracellular source of ROS production in the electron transport chain (ETC) of oxidative phosphorylation. Respiratory chain complexes I and III are the major sites of ROS generation in mitochondria [47–49]. During oxidative phosphorylation, the respiratory chain complexes transfer electrons to oxygen, mainly producing superoxide radicals and subsequently hydrogen peroxide (H_2_O_2_) and hydroxyl radicals [49, 50]. This production of ROS can be detoxified by cellular defense systems, including mitochondrial superoxide dismutase, manganese superoxide dismutase (MnSOD), glutathione peroxidase, catalase, and glutathione (GSH) [51–53]. When the balance of ROS production and antioxidant defense is perturbed, ROS accumulate and result in oxidative damage to the target cells. Cellular redox homeostasis is tightly regulated by p53 through transcription and modification of pro-oxidant and antioxidant protein [54, 55]. Various forms of cellular stress activate p53 to inhibit ROS generation and promote cell repair or to increase cellular oxidative damage and induce senescence or apoptosis under conditions of severe, irreversible stress [12]. Numerous studies have revealed that the levels and activity of p53 are substantially increased in various neurodegenerative conditions, accompanied by oxidative damage of macromolecule proteins and DNA [56–58]. Overexpression of p53 transactivates a series of pro-oxidative genes, including p53-inducible gene 3 (PIG3), p66shc, and proline oxidase gene associated with ROS production [54, 59–62]. PIGs activation, for example, causes oxidative damage of target cells through increased ROS production via NADPH-quinone oxidoreductase and inhibition of ROS scavenging by catalase [63, 64]. p53 affects mitochondrial respiratory activity by regulating the synthesis of cytochrome c oxidase 2 (SCO2). SCO2 is a nuclear DNA-encoding subunit, which is essential for regulating the cytochrome c oxidase (COX) complex, the major site of oxygen utilization in eukaryotic cells. p53 transactivates the expression of SCO2 by binding its promoter in nuclear DNA, resulting in ROS production [65]. Moreover, p53 following cellular stress induces the expression of proapoptotic proteins including B-cell lymphoma 2 (Bcl-2)-associated X-protein (Bax), p53 upregulated modulator of apoptosis (PUMA), and nicotinamide adenine dinucleotide phosphate oxidase activator (NOXA), which disturb mitochondrial function resulting in upregulation of ROS generation [66]. In addition, cytosolic p53 decreases the ubiquitin-mediated degradation of *α*-synuclein protein [67]. *α*-Synuclein targets mitochondria to induce profound mitochondrial alterations, including collapse of transmembrane potential, impairment of respiratory chain complexes, disturbance of mitochondrial Ca^2+^ homeostasis, and, finally, ROS production and oxidative stress [68–70]. Accumulation of p53 in the mitochondrial matrix binds and inactivates MnSOD, a critical mitochondrial enzyme, involved in cellular defense against oxidative stress by scavenging ROS [71]. p53 overexpression also impairs mitochondrial morphology, resulting in decreased mitochondrial Ca^2+^ transients, followed by ROS production [72]. (Figure 1). The mitochondrial ETC is a primary cellular target of ROS-induced oxidative stress, and oxidative damage leads to further inhibition of the ETC and excessive ROS production [73]. Thus, a vicious pathogenic cycle develops between the defects in ETC and ROS generation, which may be critical in the progressive loss of dopaminergic neurons and the development of PD [74]. p53 plays an essential role in these processes and provides a potential target for therapeutic intervention.

The NS dopaminergic neurons are vulnerable to oxidative stress. Increased iron levels have been detected in the SN of PD patients compared to healthy controls [75]. Iron promotes the generation of highly reactive oxygen species, resulting in further oxidative damage. DNA oxidative damage in vulnerable dopaminergic neurons is a hallmark of PD [76, 77]. Proliferating cell nuclear antigen (PCNA) is an essential protein that protects DNA from oxidative damage by regulating a wide range of enzymes and regulatory proteins [78, 79]. p53 is an upstream regulator of PCNA, and high concentration of p53 reduces the expression levels of PCNA by inhibiting its promoter, which diminishes its ability to protect DNA from oxidative damage [80,81]. Consistent with these reports, our previous studies in MPP^+^-induced neuronal PC12 cells suggested that PCNA downregulation caused by p53 activation contributed to the DNA oxidative damage in dopaminergic neurons [74]. This evidence supports the conclusion that p53 functioning as a converging signal for the generation of ROS plays a crucial role in PD pathogenesis.

### 3.2. p53 and Mitochondrial mPTP

p53 in response to cellular stress undergoes posttranscriptional modifications that increase its stabilization and subcellular translocation [29]. Nuclear translocated p53 binds to specific response sequences in the target genes and induces the expression of many proapoptotic proteins, such as Bax, PUMA, NOXA [82–84]. These proteins are essential for forming the mitochondrial permeability transition pore (mPTP) and inducing mitochondria-mediated intrinsic cell death under pathological conditions [38, 74]. Bax and Bcl-2 antagonist/killer (Bak) are proapoptotic proteins involved in mPTP formation. The antiapoptotic Bcl-2 family proteins Bcl-2 and B-cell lymphoma-extra large (Bcl-xL) combine with Bak to counter their proapoptotic function under normal conditions. Activation of p53 following cellular stress interacts with Bcl-2/Bcl-xL and releases Bax/Bak to open mPTP, leading to the release of cytochrome c from the mitochondria into the cytosol [85]. Mitochondrial translocation of p53 can directly bind Bax/Bak to disrupt the protein complex and activate the intrinsic apoptotic pathway [86,87].

p53 transcriptionally activates the proapoptotic protein PUMA [88]. Activation of PUMA binds all of the antiapoptotic BCL-2 members and facilitates Bax/Bak-mediated permeabilization of the outer mitochondrial membrane (OMM), resulting in the release of cytochrome c and activation of the caspase cascade [14]. PUMA also induces the release of cytosolic p53 from BCL-xL to activate Bax and Bak [89].

**Figure 2:**
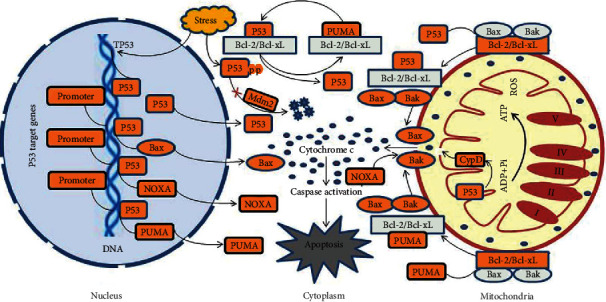
Association of p53 activation with mitochondria-mediated cell death. p53 is a response factor and becomes activated in response to cellular stress. Activation of p53 transactivates the expression of many proapoptotic proteins, including Bax, PUMA, and NOXA, which induces the opening of mPTP and initiates the mitochondria-mediated intrinsic cell death. p53 in response to cellular stress undergoes posttranscriptional modifications to control its activity and subcellular localization and to resist proteasomal degradation by Mdm2. Mitochondrial Bax and Bak are essential proapoptotic proteins that are combined with the antiapoptotic Bcl2 protein under normal conditions. Activation of p53 interacts with Bcl-2/Bcl-xL and releases Bax/Bak to opening mPTP, leading to the release of cytochrome c from the mitochondria into the cytosol and subsequent caspase activation. p53 transcriptionally activates proapoptotic protein PUMA to induce mPTP formation. PUMA contributes to the release of p53 from Bcl-xL complex into cytosol, resulting in p53-dependent Bax/Bak activation and subsequent permeabilization in the outer mitochondrial membrane. PUMA also directly interacts with proapoptotic proteins Bax and Bak to open mPTP and release cytochrome c. NOXA is a p53 target protein that impairs mitochondria via mPTP triggering intrinsic cell death. Mitochondrial p53 also interacts directly with CypD to open mPTP and trigger apoptosis.

In addition, p53 induces the expression of the apoptotic regulating factor NOXA, which facilitates the opening of mPTP and release of cytochrome c to trigger cell death [90]. Besides OMM permeabilization, p53 mitochondrial translocation also induces the opening of the permeability transition pore in the inner mitochondrial membrane (IMM) by activating the translocation of cyclophilin D (CypD) from the mitochondrial matrix to the IMM. The translocated CypD interacts with the IMM protein adenine nucleotide translocator (ANT) to induce its morphological changes and subsequent formation of the ANT channel [91]. The permeabilization of outer mitochondrial membranes together with the channel formed by ANT in inner mitochondrial membranes constitutes a tunnel-like structure that causes the release of apoptotic mediators from the mitochondria into the cytosol to trigger caspase activation and eventual cell death (Figure 2). p53 has been implicated in the regulation of mitochondrial Ca^2+^ homeostasis in numerous ways. Nuclear p53 transrepresses the expression of Pten-induced kinase 1(PINK1) through binding and inactivating its promotor [9]. PINK1 physiologically regulates calcium efflux from the mitochondria via the ion exchanger, and its deficiency causes impaired Ca^2+^ efflux resulting in mitochondrial Ca^2+^ overloading [92]. Mitochondrial translocation of p53 reduces mitochondrial Ca^2+^ transients and facilitates Ca^2+^ release into the mitochondrial matrix [72]. Ca^2+^ is an essential ion for the activation of numerous mitochondrial enzymes that are necessary for mitochondrial metabolism [93]. Mitochondrial Ca^2+^ overloading has profound consequences for the cell, including defective synthesis of adenosine triphosphate (ATP), the collapse of the mitochondrial transmembrane potential, ROS production, and activation of mitochondrial mediated cell death [94]. Ca^2+^ overloading and excessive ROS production, in turn, facilitate the mPTP opening by inducing the translocation of the mitochondrial matrix CypD to the inner membrane and activating the mPTP regulator ANT [95]. Thus, p53 overexpression and subcellular translocation play a crucial role in mitochondrial apoptotic changes and subsequent neurodegeneration.

## 4. p53 with Autophagy and Protein Aggregation

Neurodegenerative disorders are characterized by the accumulation of abnormal protein and damaged mitochondria that are associated with dysregulation of either proteasomal and/or autophagic quality control systems [96]. p53 has been known to be a key regulator in autophagic response, and activation following neurodegenerative stress leads to autophagic failure and subsequent neurodegeneration [97].

### 4.1. p53 and Autophagy

Autophagy is a major intracellular process for the elimination of deleterious proteins and damaged mitochondria; dysfunctional autophagy has been linked to the pathogenesis of numerous neurodegenerative disorders, including Alzheimer's disease (AD), Huntington's disease (HD), amyotrophic lateral sclerosis (ALS), and PD [96, 98]. p53 has been increasingly recognized to be a key autophagic regulator that functions primarily through transcriptional effects on a wide range of downstream target genes, as well as regulation of the mTOR pathway in a transcription-dependent manner [99, 100].The differential regulation of autophagy by p53 following cellular stress is dependent on its subcellular localization, targeting genes, and stress conditions. The accumulation of p53 in the cytosol has been suggested to inhibit autophagic clearance of abnormally aggregated proteins in pathogenic conditions [67, 101, 102]. p53-associated dysfunction of autophagy is increasingly considered as a potential mechanism responsible for the degeneration of dopaminergic neurons in PD pathogenesis [9, 67, 103]. Neurodegenerative conditions induce high levels of p53 that are closely associated with the abnormal accumulation of *α*-synuclein and dysfunctional mitophagy [9].

### 4.2. p53 and *α*-Synuclein Aggregation

Neuropathologically, PD is characterized by the presence of protein inclusions termed Lewy bodies (LBs) in the vulnerable neurons of the SN [104]. The synaptic protein *α*-synuclein has been identified as the primary component of LBs [105, 106]. *α*-Synuclein is an intracellular protein normally localized in the presynaptic terminals, and aggregation and dimer formation of *α*-synuclein are caused by dysfunctional cellular proteostasis [107–109]. Aberrant *α*-synuclein accumulation and formation of LBs in dopaminergic neurons have implicated the neurodegeneration [107]. Protein aggregation disrupts cellular function, leading to the activation of cell death signals and subsequent neuron injury and death [107]. p53 is a stress response gene involved in the regulation of autophagy via diverse pathways [101, 110].

Chaperone and cochaperone systems are essential for protein folding or refolding and degradation of aggregated protein; thus, they prevent the cytotoxicity caused by aberrant protein accumulation [111, 112]. p53 regulates the functional activity of HSP70 and HSP90 chaperone and cochaperone systems in neurodegenerative conditions [67, 113]. Studies in PD cellular and animal models have shown that p53 activation increases the aggregation of *α*-synuclein in vulnerable neurons through inhibiting HSP70-mediated protein folding activity, accompanied by BAG5 protein overexpression [113]. BAG5 is an important stress-induced backup nucleotide exchange factor of HSP70 associated with the protein activation. High levels of BAG5, however, inhibit the folding activity of the HSP70 chaperone, resulting in dysfunction of protein folding and refolding and subsequent abnormal protein aggregation. BAG5 expression is transcriptionally regulated by the p53 gene under stress conditions [113]. The gene silence of p53 causes a substantial decrease in BAG5 mRNA and protein levels in the stressed cells. Mechanism studies reveal that p53 can directly bind to the promoter and activate BAG5 transcription, leading to elevated levels of BAG5 under irreversible stress conditions [113]. p53 activation induces overexpression of BAG5 to inhibit the protein folding activity of HSP70, leading to the aggregation and accumulation of *α*-synuclein and subsequently cell toxicity and death.

c-Abl is a critical tyrosine kinase associated with the accumulation of pathogenic *α*-synuclein and neurodegeneration in PD [114–116]. c-Ab1 is activated in response to cellular stress, including oxidative stress and DNA damage [67]. Activation of c-Ab1 directly phosphorylates *α*-synuclein or decreases its autophagic degradation [116, 117]. Pharmacological inhibition of c-Ab1 has been shown to reduce *α*-synuclein levels or its aggregation via the activation of autophagy in PD cellular and animal models [115]. Several lines of evidence have suggested that c-Abl-dependent inhibition of autophagy also involves p53 activation and p53-dependent mTOR signal pathway [67]. c-Abl directly phosphorylates Mdm2, decreasing its ligase activity [118]. Mdm2 is a key E3 ligase that ubiquitinates p53 for proteasomal degradation and prevents p53 transcription by binding to its N-terminal domain [119]. Decreased levels and activity of Mdm2 cause the accumulation of p53 under stress conditions [120]. Studies in PD cellular and animal models have demonstrated that pharmacological inhibition of p53 can block *α*-synuclein aggregation and autophagy defects caused by c-Ab1 activation. These results support the conclusion that c-Ab1 mediates the accumulation and aggregation of *α*-synuclein, which at least in part occurs through the p53-dependent pathway under neurodegenerative conditions.

## 5. p53 and Mitophagy

Mitophagy is a protective mechanism for mitochondria to maintain their homeostasis through clearance of damaged mitochondria or fission-fragmented mitochondria via lysosomal degradation [121]. This protective function is crucial for neuronal cells due to the sensitivity of neurons to toxic aggregation. Mitophagy impairment causes the accumulation of defective mitochondria resulting in toxicity to the vulnerable neurons and eventually neuronal degeneration, and this cell death pathway has been underlined in the pathogenesis of neurodegenerative disorders, including PD [122, 123]. PINK1 and Parkin have been suggested to play a crucial role in the process of mitophagy [121]. PINK1 is a serine/threonine kinase possessing a mitochondrial targeting sequence, which allows the kinase to enter into the mitochondria and translocate to the IMM. The mitochondrial translocated PINK1 is normally cleaved and inactivated by the IMM protease presenilin-associated rhomboid-like protein (PARL) and subsequently degraded through the N-end rule pathways, resulting in low levels of PINK1 in the healthy mitochondria [124, 125]. However, mitochondrial depolarization inhibits PINK1 translocation to the IMM and subsequent degradation by PARL, which contribute to the accumulation of PINK1 on the OMM and the subsequent recruitment of Parkin from the cytoplasm into the damaged mitochondria. Parkin is an E3 ubiquitin ligase that ubiquitinates mitochondrial membrane proteins to trigger the elimination of defective mitochondria by lysosomes. The PINK1/Parkin-mediated mitophagy is crucial for mitochondrial quality control and to clean damaged mitochondria. This functional activity of PINK1/Parkin can be disturbed by p53 activation, leading to impaired mitophagy. p53 transrepresses the expression of PINK1 under normal as well as pathogenic conditions. This is supported by the finding that pharmacological phosphorylation of p53 leads to the decreased expression of PINK1 in SH-SY5Y neuroblastoma cells and inhibition of p53 activity increases both PINK1 protein expression and mRNA levels in the cell treated with pifithrin-*α* (PFT), a well-known p53 inhibitor. p53 adenoviral overexpression in mouse striatal neurons causes the decrease in PINK1 and mRNA levels, while depletion of endogenous p53 promotes its expression and mRNA levels, supporting p53 as a transcriptional inhibitor of PINK1 transcription [9]. p53 also directly interacts with Parkin to inhibit its translocation to the damaged mitochondria, resulting in the impairment of mitophagy [126]. Parkin is shown to repress the transcription of p53, which in turn transactivates the expression of Parkin [127, 128]. This interplay could increase the expression of PINK1 since its transcription is tightly controlled by p53 and p53 repression by Parkin results in PINK1 transactivation. The interplay among p53, PINK1, and Parkin creates an intricate regulating network for elimination of defective mitochondria by mitophagy, while overexpression of p53 during neurodegenerative stress decreases PINK1 levels and inactivates mitophagic activity of Parkin, resulting in impairment of mitophagy and consequent neurodegeneration.

## 6. Conclusion and Future Perspectives

p53 is a multifunctional protein that regulates numerous diverse cellular processes through transcription-dependent mechanisms and transcription-independent actions. p53-dependent neuronal death has been mechanistically linked to the pathogenesis of many neurodegenerative disorders including PD. Activation of p53 in response to neurodegenerative stress facilitates mitochondrial dysfunction, oxidative stress, aberrant protein aggregation, and autophagy impairment. These are central events associated with the degeneration of dopaminergic neurons and fundamental processes in the pathogenesis of PD. p53 plays a significant role in neurodegeneration through the integration of various neurodegenerative signals triggering neuronal death, making it a potential target for the treatment of PD. Strategies to inhibit the high levels and activity of p53 could inhibit the progression of pathological changes and alleviate the progressive degeneration of dopaminergic neurons in PD. In particular, Mdm2 binds to the transactivation domain of p53, inhibits its transcriptional activity, and mediates p53 ubiquitination and degradation via proteasomes. Pharmacological stimulation of Mdm2 has been shown to decrease p53 activity and levels and promote neuronal survival under neurodegenerative conditions. Therefore, Mdm2 appears to be a potential therapeutic target that could be used in the development of novel neuroprotective strategies for PD. In conclusion, p53-dependent therapeutic intervention is needed.

## Abbreviations

PD: Parkinson's disease
ROS: Reactive oxygen species
SN: Substantia nigra
Mdm2: Murine double minute-2
Bax: Bcl-2-associated X-protein
PUMA: p53-upregulated modulator of apoptosis
NOXA: Nicotinamide adenine dinucleotide phosphate oxidase activator
Bak: BCL-2 antagonist/killer
mPTP: Mitochondrial permeability transition pore
Bcl-2: B-cell lymphoma 2
CypD: Cyclophilin D
ATP: Adenosine triphosphate
HNE: 4-Hydroxyl-2-nonenal
H_2_O_2_: Hydrogen peroxide
MnSOD: Manganese superoxide dismutase
SCO2: Cytochrome c oxidase 2
COX: Cytochrome c oxidase
ETC: Electron transport chain
PCNA: Proliferating cell nuclear antigen
LBs: Lewy bodies
6-OHDA: 6-Hydroxydopamine
PINK1: Pten-induced kinase 1
Bcl-xL: B-cell lymphoma-extra large.
